# Supplementary material for: Spectral graph theory of brain oscillations
Source: Hum Brain Mapp. 2020 Mar 23;41(11):2980–98. doi: 10.1002/hbm.24991 (PMC7336150; doi:10.1002/hbm.24991)
Supplement: Supplementary file 1 — Table S1 Parameters values and limits [file HBM-41-2980-s001.docx]

**Supplementary Table 1: Parameters values and limits**

|  | **Initial Value** | **Lower/Upper Boundary** |
| --- | --- | --- |
| Time constants {$\tau_{e},\tau_{i},\tau_{G}$} | 12, 3, and 6 ms, respectively | [5ms, 20ms] |
| Gains {$g_{ei},g_{ii}\}$ | 4 and 1 respectively | [0.5, 5] |
| Transmission velocity $v$ | 5 m/s | [5 m/s, 20 m/s] |
| Alpha $\alpha$ | 1 | [0.1, 1] |
